# Trueness of vat-photopolymerization printing technology of interim fixed partial denture with different building orientation: A Microcomputed tomography study

**DOI:** 10.4317/jced.61422

**Published:** 2024-04-01

**Authors:** Mohamed Radwan, Ahmed Abdou, Ahmed Tawfik, Paul Bills, Liam Blunt, Citra Kusumasari, Aliaa Mahrous

**Affiliations:** 1 Fixed Prosthodontics Department, Faculty of Dentistry, Bani-Suef University, Bani-Suef, Egypt; 2 Faculty of Dentistry, Al-Ayen Iraq University, Thi-Qar, Iraq; 3 EPSRC Future Advanced Metrology Hub, University of Huddersfield, Huddersfield, United Kingdom; 4 Department of Conservative Dentistry, Faculty of Dentistry, Universitas Indonesia, Jakarta, Indonesia; 5 Fixed Prosthodontics Department, Faculty of Dentistry, Fayoum University, Fayoum,and October University for Modern Sciences and Art, 6th of October City, Egypt

## Abstract

**Background:**

The aim was to assess the consequence of different printing orientation on the marginal misfit and internal gap of 3-unit interim fixed partial denture manufactured by two different additive manufacturing technologies compared to milling technique.

**Material and Methods:**

Three-unit interim fixed partial denture (FPD) was designed by using exocad software (Dental CAD 3.0 Galway) in the format of standard tessellation language (STL) , which was transferred to a nesting software (PreForm) and printed by A Next Dent C&B resin liquid (NextDent; Soesterberg, Neitherland) by using two printing technologies; stereolithography (SLA, n=30) and digital light processing (DLP, n=30) with 3 different orientations (occlusal direction [0°] ,buccal direction [90°] & lingual direction [270°]) for each technology (n=10). Additionally, a control group was milled (CAD/Milling, n=10) from DC PMMA A1 Disc (White peaks dental solutions; Gmbh& co., Germany). A Microcomputed tomography was used to measure the marginal misfit and internal gap for each specimen in 12 different points. The average value of the marginal and internal gaps measurements was calculated, and one-way ANOVA was used for the comparison between groups.

**Results:**

SLA printing technology showed a similar result to CAD/Milling with all different printing orientations tested. DLP printing technology showed the highest gap values within all the printing orientations with significant difference (*p*< 0.001) with the CAD/Milling and SLA.

**Conclusions:**

Regarding the trueness of the interim FPDs, SLA was a promising technology for its superior adaptation. Marginal misfit and Internal gap for DLP printing technology limiting the use of that technology as it exceeded the acceptable clinical range.

** Key words:**3D Printing, Microcomputed topography, Marginal Gap, Internal Misfit.

## Introduction

The advent of 3D printing technology has revolutionized dental rehabilitation, particularly in the fabrication of interim FPDs. Interim prostheses play a vital role in enhancing occlusion equilibrium, esthetics, and function during a specified timeframe of up to six months (1). Achieving precise marginal and internal fit in these interim 3D-printed restorations is of utmost importance, especially in cases of prolonged esthetic and occlusal rehabilitation, as it significantly influences the overall outcome. The marginal gap refers to the space between the restoration margin and the finish line of the prepared tooth while the internal gap refers to the spacing between the internal surface of the restoration and the prepared tooth structure (2,3). A proper marginal and internal fit ensure the structural durability of interim FPDs and promotes the health of adjacent soft tissues, ultimately contributing to the long-term success of the restorations (4-6)

In the realm of prosthetic dentistry, the use of Computer-Aided Design and Computer-Aided Manufacturing (CAD/CAM) technology has led to the production of reliable and accurately dimensioned restoration components in a shorter time compared to conventional methods (5-8). Among CAM technologies, milling is widely considered the standard after continuous advancements over the past decades (7,9-11). Alternatively, additive manufacturing technologies have gained prominence in dentistry, including Vat-photopolymerization, which encompasses Stereolithography (SLA), Direct Light Processing (DLP), and Liquid Crystal Display-based printers(7,9,10,12-14).

Vat-polymerization technology depends on 3D photopolymerization of a layer of liquid resin to build up/down a specific object upon exposure to a light source of specific wavelength (9,10). Stereolithography (SLA) involves a layer-by-layer photopolymerization process with an ultraviolet laser beam to solidify liquid resin, offering high accuracy of approximately 50–55 µm, material flexibility, and excellent surface finish. However, it necessitates post-processing, incurs high maintenance costs, and results in prolonged printing times with limited mechanical properties (14-17). On the other hand, Direct Light Processing (DLP) uses a digital micromirror device to expose the entire resin layer at once, providing higher complexity structure printing, an accuracy of 65-70 µm, and smooth surface topography. Nonetheless, DLP has limitations in terms of build area size, printability of large parts, and buildability in the vertical direction (12,17-19).

The accuracy of additive manufacturing is characterized by trueness and precision, where trueness relates to the printer’s capability to produce a restoration precisely as per its virtual design, while precision refers to the ability to reproduce the restoration under identical conditions multiple times (14-19). Factors such as printing layer thickness, laser type, and wavelength influence the trueness of additively manufactured prostheses, and the printing direction or orientation plays a significant role in the anisotropic behavior of 3D-printed restorations (19-24).

Despite extensive research on the mechanical properties of additively manufactured prostheses, limited attention has been given to the influence of printing direction on their mechanical and physical characteristics (24-29). Moreover, no studies have investigated the optimization of printing direction on trueness values.

Therefore, this study aimed to assess the impact of printing orientation on the trueness of 3-unit interim FPDs fabricated by using SLA and DLP, and to compare them with milling technology. The primary null hypothesis posited that there were no significant differences in the trueness of 3-unit interim FPDs fabricated using different orientation techniques and diverse technologies. The secondary null hypothesis suggested that there were no noTable disparities in the marginal and internal misfit of 3-unit interim FPDs manufactured through either additive or milling technology under optimal conditions.

## Material and Methods

A three unit all ceramic FPD preparation for missing lower first molar tooth was prepared on dental typodont model (Nissin Hard Gingiva Jaw Model PRO 2001-UL-HD-FEM-32; Kyoto, Japan), by using diamond stones (837KR, Intensive SA; Montagnola, Switzerland) attached to a manual milling machine (Aciera F3 milling machine, DeguDent; GmbH, Germany). Abutment margins were prepared with a supragingival 1mm chamfer finish line thickness, while the occlusal surfaces were reduced by 1.5 mm clearance and a 6° convergence angle. An addition silicone-based impression material (Express, 3M ESPE; Seefeld, Germany) was used for preparing a negative replica record of the preparation. Then, epoxy resin (Chema poxy150 3D, CMB chemicals; Cairo, Egypt) was used to produce a master epoxy model.

The epoxy model was scanned by using benchtop 3D scanner (E4, 3Shape; Copenhagen, Denmark) with 4 µm resolution and saved in STL format. A virtual design of the interim FPD was designed by using the computer-aided design (CAD) software 3.0 Galway (Exocad; GmbH, Darmstadt, Germany), featuring a 60 µm cement space that was terminated 1 mm above the finish line. The CAD STL file of the virtual interim FPD was used to construct a total of 70 interim FPDs in all tested groups with different construction techniques.

The first group (SLA group) used Stereolithography printing technology (Formlabs Form 3B, Formlabs Inc.; MA, USA) to print FPDs with 25 μm resolution, 250 nm laser power and (145 x 145 x 185) mm build volume. The second group (DLP group) used Digital Light Processing printing technology (Rapidshape D30+; Heimsheim, Germany) with 35 μm resolution, 385 laser power and a larger build volume (480 x 690 x 410) mm. The third group (control group) used a five-axis milling machine (Imes-icore 250i, Imes-icore GmbH; Germany) to construct interim FPDs (n=10) from Polymethyl Methacrylate (PMMA A1 Disc, White peaks dental solutions; Gmbh & co., Germany).

**Figure 1:**
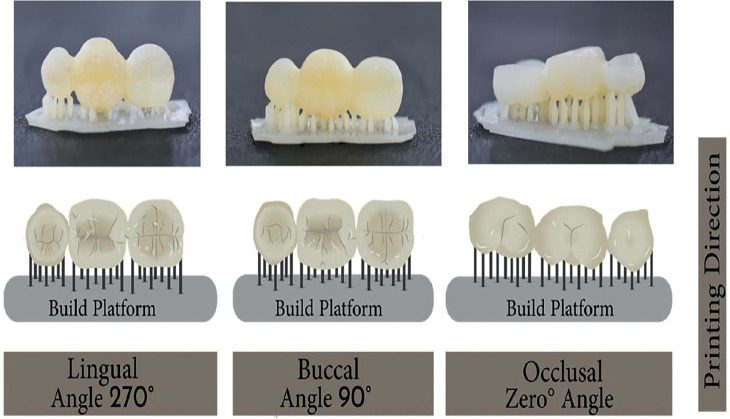
Interim fixed denture with different Printing Direction. A. lingual orientation building direction ( 270°) , B. occlusal orientation building direction (90°) , C. Buccal orientation building direction (0°).

SLA and DLP groups were using a liquid resin (Next Dent C&B, Next Dent; Soesterberg, Neitherland) as a printing media in 3 different orientations (Occlusal, Buccal, and Lingual). Occlusal direction (n=10 for each technology): the liquid resin material was oriented with the occlusal surface facing the build platform with 50 µm layer thickness and printing angle of 0° (23). The buccal direction (n=10 for each technology): the liquid was oriented with the buccal surface facing the build platform with 50 µm layer thickness and printing angle of 90°. Lingual direction (n=10 for each technology), which was same as buccal but the lingual surface facing the building platform and printing angle was 270° (Fig. 1).

After complete printing, all FPDs were immersed and rinsed in a tube containing isopropyl alcohol (ReAgent Chemical Services; Halton, Runcorn, United Kingdom) to remove the uncured resin. Subsequently, FPDs underwent cleaning through an ultrasonic bath (hygea 2850VM, Ultrawave Ltd; Wentloog, Cardiff, UK). Following the cleaning process, supports removal were carried out by using flush cutters (Wire Cutters UK; Clitheroe, Lancashire, UK). Then, the produced FPDs were post-cured for 30 min with an ultraviolet light curing unit (Next Dent LC-3D Print Box, NextDent; Soesterberg, Neitherland), to ensure the full polymer conversion. The post curing cycle lasted for 30 minutes with blue UV light source with 315-400 nm wavelength and a power of 72 watt. This procedure was a necessary step to produce a biocompatible end-product with the highest mechanical properties of the polymer. All milling and printing FPDs were finished and polished with stone (Jota Arkansas stone 649, Jota; Ruthi, Switzerland) to get a smooth surface. Ethanol solution was used for cleaning of the FPDs. All fabricated interim FPDs were then placed on the master die to be checked with magnification loups for complete seating verification.

An epoxy model was fixed to a jig to ensure the same scanning position during micro-CT (µCT) scanning. The FPDs were then seated on the epoxy model without cementation. Each FPD was scanned with a µCT (Nikon MCT 225; Nikon Tring, UK) that was calibrated prior to scanning with a standard 5 ruby artefact and the operating condition for the µCT device was 250µm filter, 4000ms exposure, 7.6 W Filament Current, 170 KV Acceleration Voltage and 9.2 µm voxel size.

**Figure 2:**
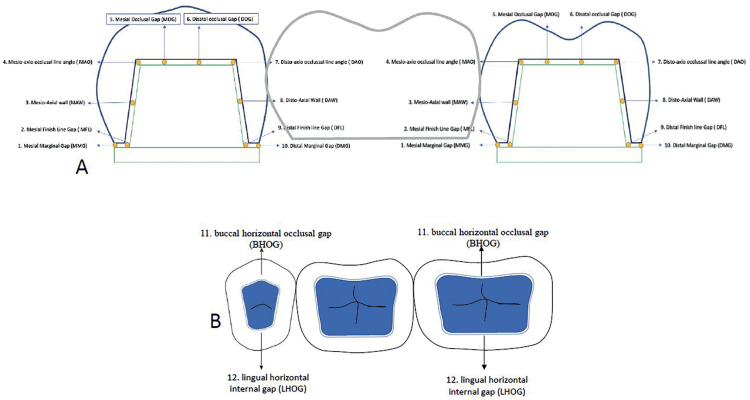
A. Schematic Diagram of the sagittal view ( Mesio-Distal ). B. A horizontal view for Micro-CT measuring point for the marginal and internal adaptation.

All µCT scanning files were reconstructed by using 3D simulation software (VGStudio MAX 3.4; Volume Graphics GmbH), then imported into a 3D data analysis and visualization software (VG studio max 3.4, Volume Graphics, Germany). A horizontal plane containing reference points from 1 to 6 were defined as a reference plane. A section perpendicular to the reference plane including points 1 and 2 was defined as a coronal section (Fig. 2). A section perpendicular to the reference plane including points 3 and 4 was defined as a premolar sagittal section. A section perpendicular to the reference plane including points 5 and 6 was defined as a molar sagittal section (Fig. 2). Marginal fit and internal gap length were measured in coronal section, premolar sagittal section, and molar sagittal section, respectively.

**Figure 3:**
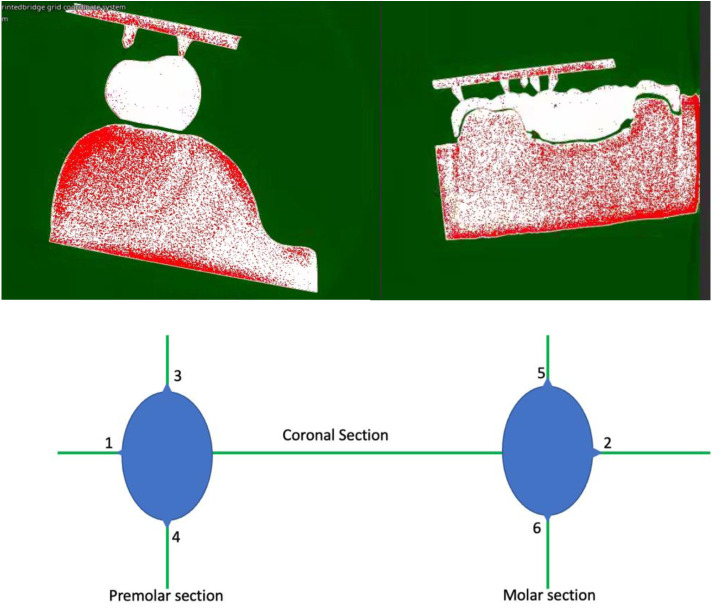
A horizontal plane containing reference points from 1 to 6 for the µCT measuring reference.

**Figure 4:**
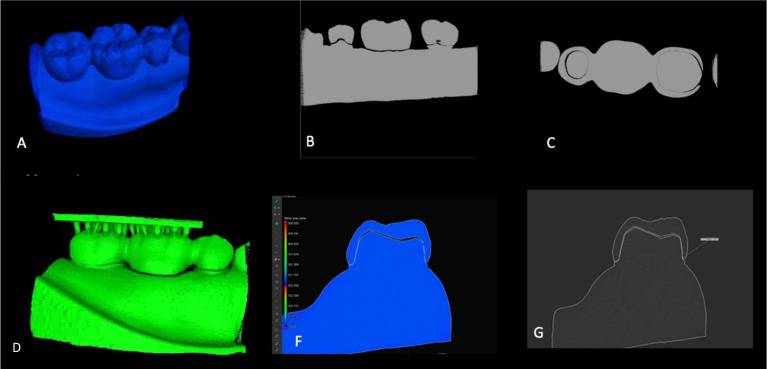
Micro-CT evaluation of interim FPD. A: seating evaluation of interim FPD, B: sagittal section evaluation, C: horizontal plan evaluation, D: marginal fit evaluation, F: internal fit evaluation, and G: Finish line measurement.

A step-by-step samples measurement for 3D printed and milled FPDs with a cross-sectional layer image was obtained from the constructed 3D scan and ten equidistant points defined from mesial to distal sides were used for the measurements (Figs. 3,4), which were calculated with the Measurement-3D distance Length tool value in microns. The µCT result analysis was carried out with a fixed grey value threshold ISO 50% for all the scans and used for all the specimens, by a single operator.

Marginal & Internal gap lengths were measured at the following points: marginal gap length four points were measured for each abutment with total eight points for each FPD [4 points for each abutment]; Mesial marginal Gap (MMG), Mesial Finish line gap (MFL), Distal marginal Gap (DMG), and Distal Finish line gap (DFL). Internal Gap length was measured with six points for each abutment in sagittal section; (mesio-axial wall gap (MAW) was measured at the midpoint of the mesial axial wall, mesio-axial occlusal line angle gap (MAO),mesial occlusal gap (MOG)) was measured at the midpoint between the center of occlusal plane and the mesio-axio occlusal angel, Distal Occlusal gap (DOG) ) was measured at the midpoint between the center of occlusal plane and the disto-axio occlusal angel, Disto-axio occlusal line angle gap (DAO) ,Disto-axial wall gap (DAW) was measured at the midpoint of the distal axial wall & last two points were in horizontal plan; Buccal horizontal occlusal gap (BHOG) at the midpoint of the buccal wall, lingual horizontal occlusal gap (LHOG) at the midpoint of the lingual wall. All measurements were taken at a magnification of 100×, and each measurement was repeated three times, and the mean value was used.

Statistical analysis:

The data was explored for normality using Kolmogorov-Smirnov and Shapiro-Wilk tests. This indicated that the data showed normal distribution, so one-way ANOVA was then used to show the effect of different tested groups on the mean gap formed followed by Tukey’s HSD for pairwise comparison. The significance level was set at *p*<0.05. Statistical analysis was performed with SPSS (IBM SPSS Statistics for Windows, Version 26.0. Armonk, NY: IBM Corp.).

## Results

Size of gap formed at the marginal, internal surfaces and results of one-way ANOVA are presented in Tables 1-2. For marginal misfit and internal gap (Table 1), one-way ANOVA showed that different groups have a significant effect on the gap formed at *p* < 0.001. For both surfaces, the statistical analysis suggested that there was an insignificant difference in results between CAD/Milling and STL with different printing directions (occlusal, buccal, and lingual). DLP/lingual showed the highest gap values followed by DLP/buccal followed by the smallest gap for DLP/Occlusal. Although DLP/Occlusal showed the lowest gap values among other DLP printing directions, the gap formed still higher than STL and CAD/Milling.

**Table 1:**
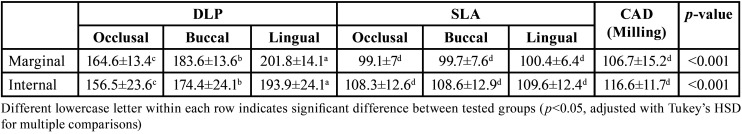
Mean, standard deviation (SD) of gap at marginal surface and internal surface for different tested groups.

The gap formed for each point in the fitting and marginal surfaces were presented in Table 2. For all points, DLP/Lingual showed the highest gap compared to all other groups.

**Table 2:**
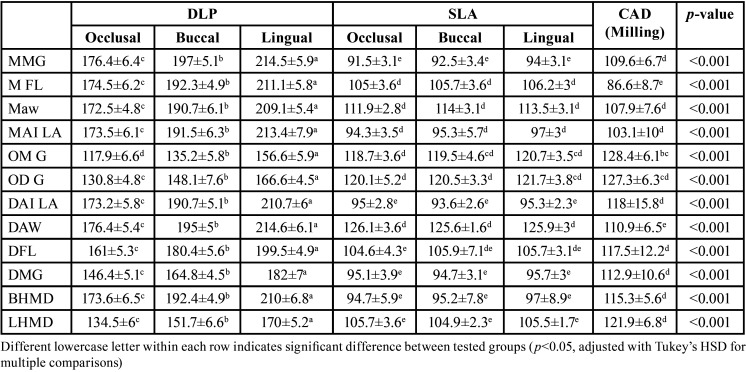
Mean, standard deviation (SD) of each tested fitting points for different tested groups.

## Discussion

Having appropriate marginal and internal fit of interim FPDs is essential for long term successful restorations. Interim FPDs may provide protection of the prepared teeth, promoting proper healing and function of the surrounding tissues, achieving a natural and esthetically pleasing appearance, with improving patient comfort. In addition to prevent bacterial invasion of the abutments and decrease post-operative sensitivity. This study aimed to assess the impact of printing orientation on the trueness of 3-unit interim FPDs fabricated by using SLA and DLP, and to compare them with milling technology. The primary null hypothesis was rejected as a significant difference in the trueness of 3-unit interim FPDs fabricated using different orientation techniques and diverse technologies. The secondary null hypothesis which suggested that there were no noTable disparities in the marginal and internal misfit of 3-unit interim FPDs manufactured through either additive or milling technology under optimal conditions was also rejected

In the present study, the trueness of the restorations was measured by using Micro-computed tomography, a non-invasive imaging technique that employed X-rays to create high-resolution 3D images of various samples. Micro-CT was gaining popularity across different fields, such as biomedical research (30) as it provided both quantitative and qualitative measurements. Micro-CT images are instrumental in assessing restoration or die quality and identifying structural defects or imperfections (26,27). It offers the advantage of measuring any angle or site, providing high-quality 2D or 3D dimensional images. However, micro-CT has many limitations as it is a costly tool and can only be used in measurements of in-vitro or experimental animal studies till now. The accuracy of measurements can be affected by beam hardening artifacts, which arise when X-rays are absorbed more by denser areas of the material, resulting in dark streaks or bands in the image. Additionally, it relies on variations in X-ray attenuation to generate contrast between different materials, and this can be limited to some materials. Also, image stitching may be required for large samples with subsequent measurement errors. For this experiment, an epoxy die, and printing resin were used for their suitability in Micro-computed tomography analysis due to the optimal difference in density and refractive index between the two materials, along with an empty cement space (23,27).

Besides, marginal gaps were measured under FPDs without cementation in order to avoid the radiation artifacts and the inappropriate difference in radio-opacity that complicating gap measures. As well, it was reported that the cement material may interfere with the complete seating of the FPDs and can prevent proper marginal sealing (31,32). Unkovskiy *et al*. (30) reported that the marginal gap before cementation in FDP was 53 µm and increased to 63 µm after cementation. Reymus *et al*. (33) also reported that the marginal discrepancy of the crown increased from 96 um to 130 um after cementation. Further researches are required to analyze the effect of luting agents on marginal gaps of 3D printed prosthesis.

The first null hypothesis was rejected as the first null hypothesis was rejected, indicating that the different printing orientations and technologies had an impact on the marginal misfit and internal gap of the interim fixed partial dentures.

The first and the second null hypothesis were rejected, indicating that there were significant differences in the trueness of 3-unit interim fixed partial dentures (FPDs) fabricated using different orientation techniques and diverse additive manufacturing technologies compared to milling. As for the different construction methods, SLA group with all printing directions, showed a significantly lower marginal misfit and internal gap (Table 1) when compared with DLP group while the difference was insignificant with the milling group (control). These could be attributed to the difference in technologies as SLA employed a more precise and effective ultra-violet laser beam which cures the resin layer point by point building a packed, precise, and dense layer with better printing quality and accuracy. While DLP used ultra-violet light source that cures a complete layer of resin at each time resulted in high printing speed with large building platform (9). Consequently, SLA is beneficial in printing precise restorations with sophisticated structure while DLP is valuable in the short printing time for finer parts with fewer details (7,9,29,34) and marginal gap values higher than the clinical accepTable range (120 µm) (23,27).

Regarding the different printing orientations, marginal misfit and internal gap revealed a significant difference among DLP group (Table 2). This result was tighter than that reported from Cho *et al*. (35) who showed a mean internal gap varies as a function of the space between 90 µm and 227 µm. The printing orientation changes from the basic site 0°(occlusal surface building orientation) to 900 & 2700 (buccal surface and lingual surface orientation) increased the unsupported surface areas leading to building orientation with large unsupported occlusal surface area. This, in turn, had an impact on the printing accuracy leading to decrease the printing precision and trueness as described by the Alharbi *et al*. (22). This was in accordance with Park *et al*. (24) who reported that the restoration quality will vary greatly due to the change of the support direction from the 00 occlusal surface orientation to any other orientation. Arnold *et al*. (36) also, reported that the re-assembled printing shape caused sagging due to plasticity phenomena and gravity, this shape is greatly changed with different orientation. Additionally, the DLP 3D printer polymerizes one layer at a time, so If the shape of the layer changed by build orientation, the form and degree of polymerization shrinkage would change (27,37).

As for SLA group, the results showed insignificant difference between the different building orientation (Table 1), these results were consistent with previous studies (29,38) that discussed the effect of changing the printing orientation from perpendicular to parallel (0° , 90° or 2700 positions) in relation to the building platform that may led to a higher intra-layer strength compared with the inter-layer strength (7,9,10,12,13) giving more precise restoration and questionable mechanical properties (4,27). The higher intra-layer strength was previously explained via point-by-point resin building curing (9). Undoubtedly, SLA printing will suffer from sagging also, especially in large or complex parts that can be affected by printing orientation of the parts, the printing layer thickness, and the curing time of the resin. Careful consideration of these factors, along with the use of support structures, could help minimize sagging and produce high-quality SLA printed structure. However, Park *et al*. (24) concluded that the difference between 3-unit interim FPD printed with 0° and 90° orientations was non-significant. while other studies (20,24,34) suggested that bar samples printed in a 90° buccal surface printing orientation were the most accurate. Contrary, Two recent studies (7,31) suggested that a build direction of 270° lingual surface orientation offered the lowest deviation for 3D-printed casts because the concave lingual surface is facing down and is supported by the build plate, which helps to minimize the amount of sagging that can occur during the printing process. Another difficulty related to SLA is the layer polymerization, as it was previously observed that samples printed with this technology appears to have lower degree of conversion at the “top” (near the printing platform) then at the “base,” which in the case of this study could possibly influence on the marginal misfit property of the provisional restorations (7,24,33,39,40), a topic that can be further investigated in the future.

Generally, the marginal misfit and internal gap of the 0° printing orientation of either DLP or SLA of the interim FPDs showed the least results (Table 2). These could be explained by many factors as the difference in the self-supporting surface, plasticity, gravity, amount of support generation, and geometry or shape of the prosthesis (35,37,39,40). In addition, the accuracy of the 3D printer in the z-axis differs from the accuracy of the other axes (35). For these various reasons, build orientation greatly influenced the marginal and internal gap and subsequently the trueness of the restorations. This difference in the result of the printing orientations may be contributed to the difference in the printing technology between the SLA and DLP (6,7,9,10) Also, depending on the build orientation, the appearance of the layer output by the 3D printer changes (20,24,37,39).

However, it was clear that none of the tested groups had mean internal or marginal gap values close to the designed cement gap of 60 µm. This could be due to various factors that can affect the accuracy of 3D printing, including the resolution of the printing technology, the resin properties, and the methods employed for post-processing techniques. However, it is important to note that the clinical significance of these discrepancies may be limited, as they are within the clinical accepTable range. While the ideal marginal and internal fit is desirable, it is often challenging to achieve in practice, and small discrepancies may not have a significant impact on clinical outcomes.

The study results showed that SLA had similar internal and marginal gap values to milled FDPs (Table 2), leading clinicians to prefer SLA due to its advantageous factors such as cost-effectiveness, design flexibility, faster production times, customization options, and access to a wide range of materials.

## Conclusions

Within the limitations of the present study.

1-SLA printed interim FPDs demonstrated excellent selection for long term temporization with minimal marginal misfit and internal gap and better trueness.

2-Printing interim FPDs with DLP technology is not recommended as the marginal gap values was exceeding the clinical accepTable range (120 µm) for interim restorations.

3-Changing the building orientation may influence the marginal and internal gap of 3D printed restorations.

4-Occlusal orientation of printed restorations may be considered as the preferred direction with better marginal and internal fit.
